# A bivalent SARS-CoV-2 monoclonal antibody combination does not impact the immunogenicity of a vector-based COVID-19 vaccine in macaques

**DOI:** 10.1126/scitranslmed.abo6160

**Published:** 2022-07-12

**Authors:** Joseph P. Nkolola, Jingyou Yu, Huahua Wan, Aiquan Chang, Katherine McMahan, Tochi Anioke, Catherine Jacob-Dolan, Olivia Powers, Tianyi Ye, Abishek Chandrashekar, Daniel Sellers, Julia Barrett, Yueh-Ming Loo, Mark T. Esser, Robert H. Carnahan, James E. Crowe, Dan H. Barouch

**Affiliations:** ^1^ Center for Virology & Vaccine Research, Beth Israel Deaconess Medical Center, Boston, MA, 02115, USA.; ^2^ Microbial Sciences, AstraZeneca, Gaithersburg, MD, 20878, USA.; ^3^ Vanderbilt Vaccine Center, Vanderbilt University Medical Center, Nashville, TN, 37232, USA.; ^4^ Department of Pediatrics, Vanderbilt University Medical Center, Nashville, TN, 37232, USA.; ^5^ Department of Pathology, Microbiology, and Immunology, Vanderbilt University Medical Center, Nashville, TN, 37232, USA.; ^6^ Ragon Institute of MGH, MIT and Harvard, Cambridge, MA, 02139, USA.

## Abstract

Human monoclonal antibodies (mAbs) that target the spike glycoprotein of Severe Acute Respiratory Syndrome Coronavirus 2 (SARS-CoV-2) offer a promising approach for the prevention and treatment of coronavirus disease 2019 (COVID-19). Given suboptimal global vaccination rates, waning immunity in vaccinated individuals, and the emergence of SARS-CoV-2 variants of concern, the use of mAbs for COVID-19 prevention may increase and may need to be administered together with vaccines in certain settings. However, it is unknown whether administration of mAbs will impact the immunogenicity of SARS-CoV-2 vaccines. Using an adenovirus vector-based SARS-CoV-2 vaccine, we show that simultaneous administration of the vaccine with SARS-CoV-2 mAbs does not diminish vaccine-induced humoral or cellular immunity in cynomolgus macaques. These results suggest that SARS-CoV-2 mAbs and viral vector-based SARS-CoV-2 vaccines can be administered together without loss of potency of either product. Additional studies will be required to evaluate co-administration of mAbs with other vaccine platforms.

## Teaser

A human SARS-CoV-2 monoclonal antibody cocktail can be co-administered with a SARS-CoV-2 viral vector-based vaccine.

## INTRODUCTION

Severe Acute Respiratory Syndrome Coronavirus 2 (SARS-CoV-2), identified in 2019 (*1*), is the etiologic agent of Coronavirus Disease 2019 (COVID-19). SARS-CoV-2 has led to over 530 million infections and more than 6.3 million deaths worldwide (*2*). Since the isolation and identification of SARS-CoV-2, several effective vaccines targeting the viral surface spike (S) glycoprotein were developed (*3*, *4*). However, global vaccine distribution challenges, waning durability over time in vaccinated individuals, and the emergence of virus variants of concern has led to the need to develop additional interventions as complementary tools to vaccines.

The development of monoclonal antibodies (mAbs) that neutralize SARS-CoV-2 offers one such promising approach for COVID-19 prevention and treatment. Such mAbs may have an increased role in the evolving pandemic, and they may need to be administered in conjunction with vaccines in outbreak settings. Indeed, several mAbs that focus on the receptor binding domain (RBD) of SARS-CoV-2 S protein have shown benefit in both pre-clinical (*5*, *6*) and clinical settings (*7*). However, an important question is whether co-administration of mAbs with vaccines will reduce vaccine immunogenicity.

Using a non-human primate model, we sought to address this gap in knowledge by investigating whether mAbs impact the immunogenicity of a prototypic viral vector-based SARS-CoV-2 vaccine. We evaluated the effect of co-administering the previously described SARS-CoV-2 human mAb cocktail AZD7442 consisting of tixagevimab (AZD8895) and cilgavimab (AZD1061) (*6*, *8*) on the immunogenicity of a vector-based rhesus adenovirus serotype 52 (RhAd52) vaccine expressing SARS-CoV-2 S protein with two proline stabilizing mutations (RhAd52-S.PP) (*9*).

## RESULTS

### AZD7442 monoclonal antibody combination shows a biphasic pharmacokinetic profile when co-administered with a SARS-CoV-2 viral-vectored vaccine

**Fig. 1.**
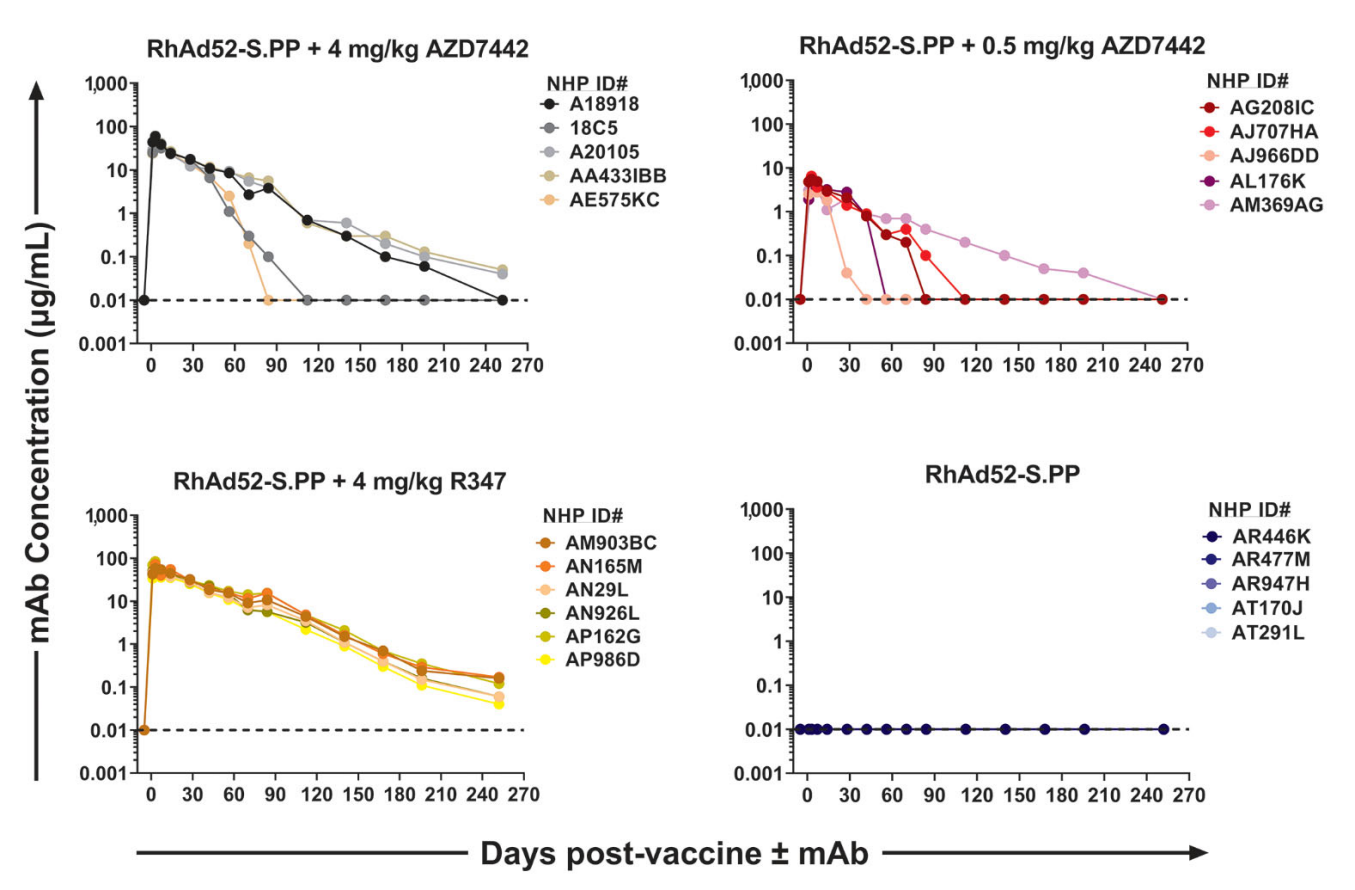
Monoclonal antibody pharmacokinetics are biphasic post co-administration with a SARS-CoV-2 viral-vectored vaccine. Antibody concentrations were determined by a human-IgG-specific ELISA. Each line correspondences to a single animal (n=6 per group). The horizontal dashed line represents assay limit of detection; A single animal was excluded from the RhAd52-S.PP + AZD7442 4 mg/kg, RhAd52-S.PP + AZD7442 0.5 mg/kg and RhAd52-S.PP groups due to high assay background observed with baseline samples. Each data point is represented by a single technical replicate. Data were generated over several batch analyses of samples (N=1 experiment).

Twenty-four cynomolgus macaques (n=6 per group) were immunized with rhesus adenovirus serotype 52 (RhAd52) expressing WA1/2020 SARS-CoV-2 S protein with two proline stabilizing mutations (RhAd52-S.PP) (*9*) by the intra-muscular route. Groups received concurrent administration of 4, 0.5, or 0 mg/kg AZD7442 mAbs or 4 mg/kg isotype matched sham mAb R347 by the intravenous route. Antibody pharmacokinetics were assessed by a human IgG specific enzyme-linked immunosorbent assay (ELISA) (*5*) and showed typical biphasic profiles of rapid serum distribution followed by slower elimination phases (**Fig. 1** **; table S1**). Peak mAb titers were generally observed on day 3 after administration and were dose-dependent. Animals administered 4 mg/kg AZD7442 exhibited a median peak concentration of 58.6 μg/mL (range 43.5 to 85.1 μg/mL). Animals administered 0.5 mg/kg AZD7442 exhibited a median peak of 5.8 μg/mL (range 4.3 to 15.6 μg/mL) with all animals having undetectable mAb titers at study conclusion. Animals receiving IgG isotype control antibody R347 at 4 mg/kg had a median peak of 63.4 μg/mL (range 55.5 to 83.9 μg/mL) and detectable mAb titers throughout the study duration. Human IgG was not detected in the group that received RhAd52-S.PP (*9*) without mAb co-administration (**Fig. 1** **; table S1**).

### AZD7442 monoclonal antibody combination exhibits varying neutralization activity against SARS-CoV-2 parental and Omicron pseudoviruses

**Fig. 2.**
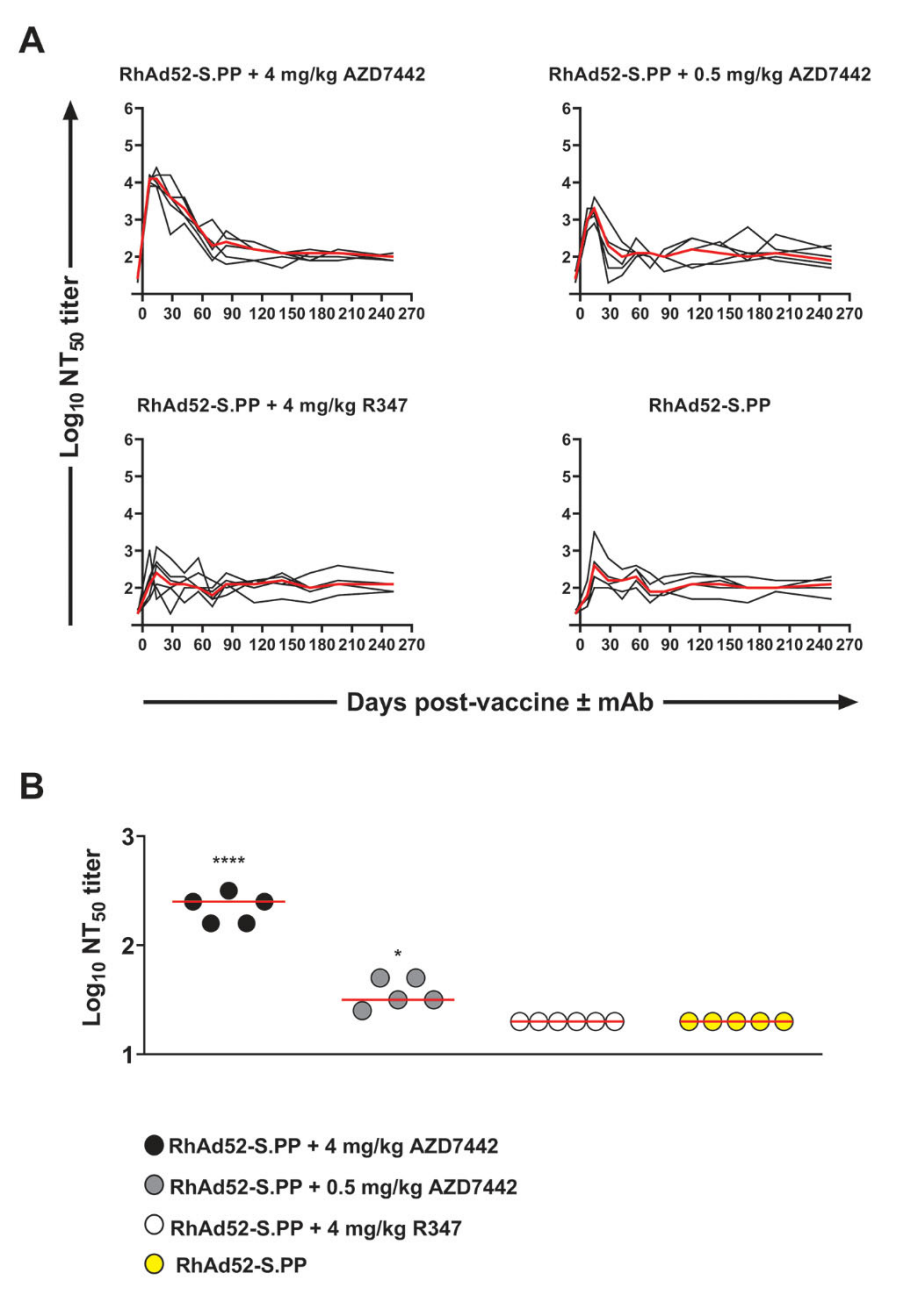
**SARS-CoV-2 pseudovirus neutralization titers are detectable post co-administration of AZD7442 mAb with a SARS-CoV-2 viral-vectored vaccine. (A)** WA1/2020 NT_50_ neutralization titers were determined by a SARS-CoV-2 lentiviral pseudovirus neutralization assay. Each line corresponds to a single animal and the red line depicts the median (n=6 per group). **(B)** B.1.1.529. NT_50_ neutralization titers were determined by a SARS-CoV-2 lentiviral pseudovirus neutralization assay. Red horizontal bars indicate median values. A single animal was excluded from the RhAd52-S.PP + AZD7442 4 mg/kg, RhAd52-S.PP + AZD7442 0.5 mg/kg and RhAd52-S.PP groups due to high assay background observed with baseline samples. Each data point is represented by a single technical replicate. Data were generated over several batch analyses of samples (N=1 experiment). A one-way ANOVA with Tukey correction was used for statistical analyses. ****P-adj<0.0005; *P-adj<0.05).

To evaluate the functional activity of the SARS-CoV-2 specific AZD7442 mAb cocktail in vivo, a lentivirus-based pseudovirus neutralization assay (*10*) against the matching SARS-CoV-2 WA1/2020 virus strain was performed. Experimental groups receiving AZD7442 at either 4 mg/kg or 0.5 mg/kg exhibited median peak half-maximal neutralizing titers (NT_50_) that were higher than those observed in the 4 mg/kg R347 or RhAd52-S.PP alone control groups (**Fig. 2A** **; table S2**). Peak NT_50_ titers were dose-dependent, concordant with the pharmacokinetic data. The 4 mg/kg AZD7442 group exhibited a median peak titer of 4.1 log10 NT_50_ (range 3.9 to 4.5), the 0.5 mg/kg AZD7742 group showed a median peak titer of 3.3 log10 NT_50_ (range 2.9 to 3.9), the 4 mg/kg R347 control group demonstrated a median peak titer of 2.4 log10 NT_50_ (range 1.7 to 3.1), and the RhAd52-S.PP alone group showed a median peak titer of 2.6 log10 NT_50_ (range 2.0 to 3.5). The latter two groups that received RhAd52-S.PP either alone or with R347 showed comparable NT_50_ profiles, reflective of vaccine-induced neutralizing antibodies (**Fig. 2A** **; table S2**). The pseudovirus neutralization assay was unable to distinguish between the AZD7442 mAbs and endogenous vaccine-induced neutralizing antibodies.

We also compared NT_50_ titers against the B.1.1.529 (Omicron) variant on Day 7. In this analysis, the AZD7442 mAb cocktail showed low but detectable NT_50_ titers to B.1.1.529. (**Fig. 2B** **; table S3**). The Omicron NT_50_ titers from the two groups that received the AZD7442 cocktail were higher than the control groups (adjusted P<0.0005, P<0.005; **Fig. 2B** **; table S3**). These data show that AZD7442 retains partial neutralization activity against the Omicron variant.

### Viral-vectored SARS-CoV-2 vaccine-induced humoral immunity is not impacted by the co-administration of AZD7442 mAb cocktail

**Fig. 3.**
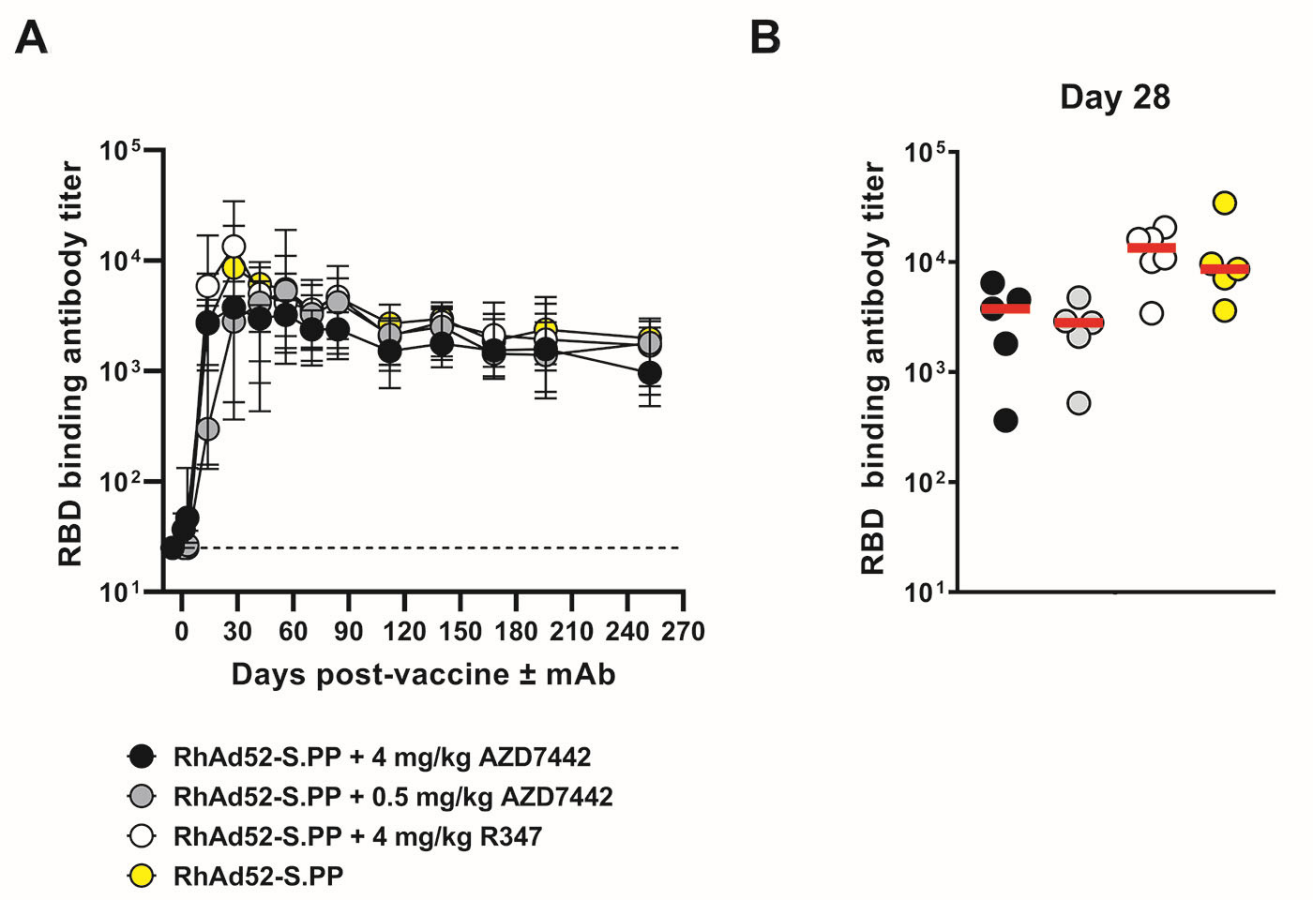
**Vaccine-induced SARS-CoV-2 RBD antibody binding titers are not impacted by AZD7442 co-administration. (A)** Longitudinal endpoint antibody binding titers to WA1/2020 RBD were determined by ELISA. Each line corresponds to an individual experimental group with data points reported as median and range (n=6 per group). Data are presented as median±range. The horizontal dashed line represents assay limit of detection. **(B)** A comparison of peak (Day 28) WA1/2020 RBD ELISA binding antibody titers is shown. Red horizontal bars indicate median. A single animal was excluded from the RhAd52-S.PP + AZD7442 4 mg/kg, RhAd52-S.PP + AZD7442 0.5 mg/kg and RhAd52-S.PP groups due to assay background observed with baseline samples. Data points used to determine median of each group were the mean of technical duplicates. Data were generated over several batch analyses of samples (N=1). A one-way ANOVA with Tukey’s correction was used for statistical analyses.

To quantify vaccine-induced antibodies and differentiate them from the infused AZD7442 mAbs, a SARS-CoV-2 WA1/2020 RBD endpoint binding ELISA assay using a secondary anti-macaque IgG antibody that did not cross-react with human IgG was used. Negligible antibody titers were observed at baseline (**Fig. 3A** **, table S4**). Macaque RBD-specific binding antibody titers were first observed in all four experimental groups at two weeks after vaccination. At peak immunogenicity (Day 28), comparable macaque RBD-specific binding antibody responses were observed across all groups with no significant (P>0.05) differences between the groups **(** **Fig. 3B** **; table S4).** These antibodies remained stable through the study conclusion at week 36 (**Fig. 3A** **; table S4**). These antibody titers represent vaccine-induced binding antibodies, and not the human mAbs in AZD7442, showing that the AZD7442 mAb cocktail did not impact the humoral immunogenicity of the RhAd52-S.PP vaccine.

### Vaccine-induced humoral immunity against SARS-CoV-2 variants is unaffected by the presence of AZD7442 mAb cocktail

**Fig. 4.**
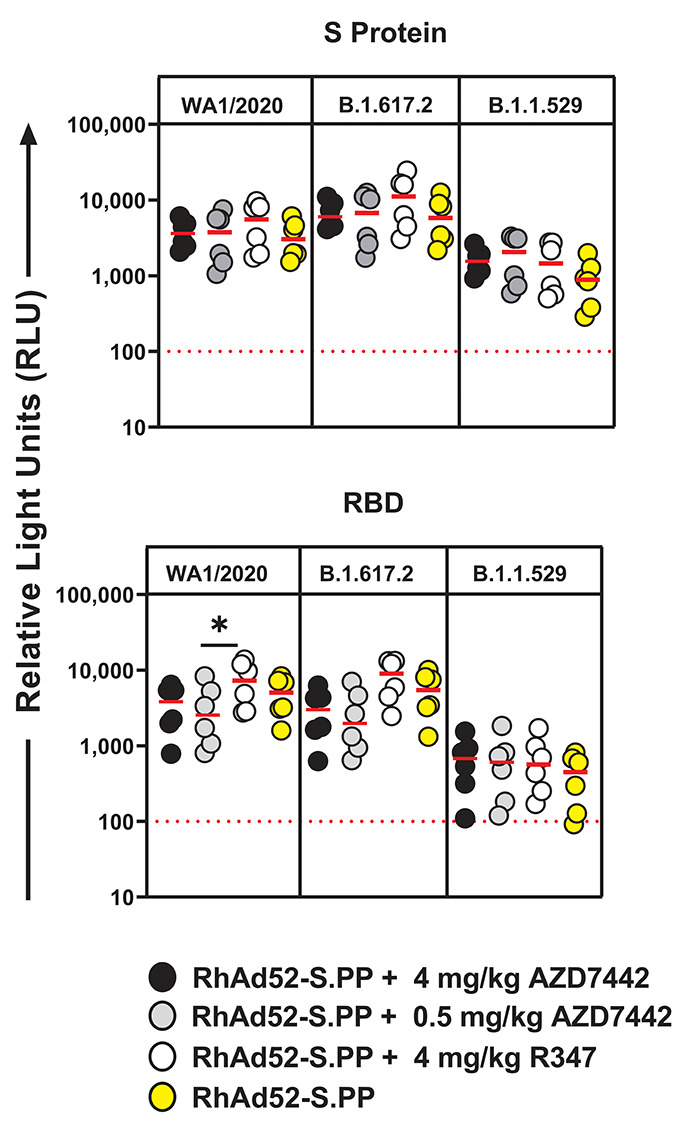
Peak vaccine-induced humoral immunity against SARS-CoV-2 S protein and RBD variant antigens is not impacted by AZD7442 mAb co-administration. Electrochemiluminesence (ECL) multiplex IgG binding responses against S protein and RBD WA1/2020, B.1.617.2 and B.1.1529 variant antigens at peak immunogenicity (Day 28) are shown. Solid red horizontal lines depict median quantities (n=6 per group). Horizontal red dotted lines depict arbitrarily-defined assay positivity threshold of mean background + 2 standard deviations. Each data point is the mean of technical duplicates. Data were generated over several batch analyses of samples (N=1 experiment). A one-way ANOVA with Tukey‘s correction was used for statistical analyses; *P-adj<0.05.

To assess whether AZD7442 impacted vaccine-induced humoral immunity against multiple SARS-CoV-2 variant lineages, multiplex IgG serology assays (Meso Scale Diagnostics) against panels of S protein or RBD variant antigens were performed at peak immunogenicity (Day 28). For this analysis, the same anti-macaque IgG detection antibody used in the endpoint ELISA that did not cross-react to human IgG was utilized. A comparison of the vaccine-induced S protein and RBD IgG binding responses against parental WA1/2020 and current, prominent circulating strains B.1.617.2 and B.1.1.529 revealed no discernable significant differences between groups receiving the RhAd52-S.PP vaccine either in the presence or absence of AZD7442 (P>0.05) **(** **Fig. 4** **; tables S5 and S6).** Binding antibody responses were also not different at peak immunogenicity against B.1.351, B.1.1.7 and P.1 S protein and RBD variant antigens (**fig. S1; table S5 and S6**). These data were concordant with the ELISA data (**Fig. 3B** **; table S4**). These data show that vaccine-induced humoral immunity against multiple SARS-CoV-2 variants was not impacted by co-administration of the AZD7442 mAb cocktail with the vaccine.

### AZD7442 mAb cocktail does not impact vaccine-induced IFN-γ cellular immunity

**Fig. 5.**
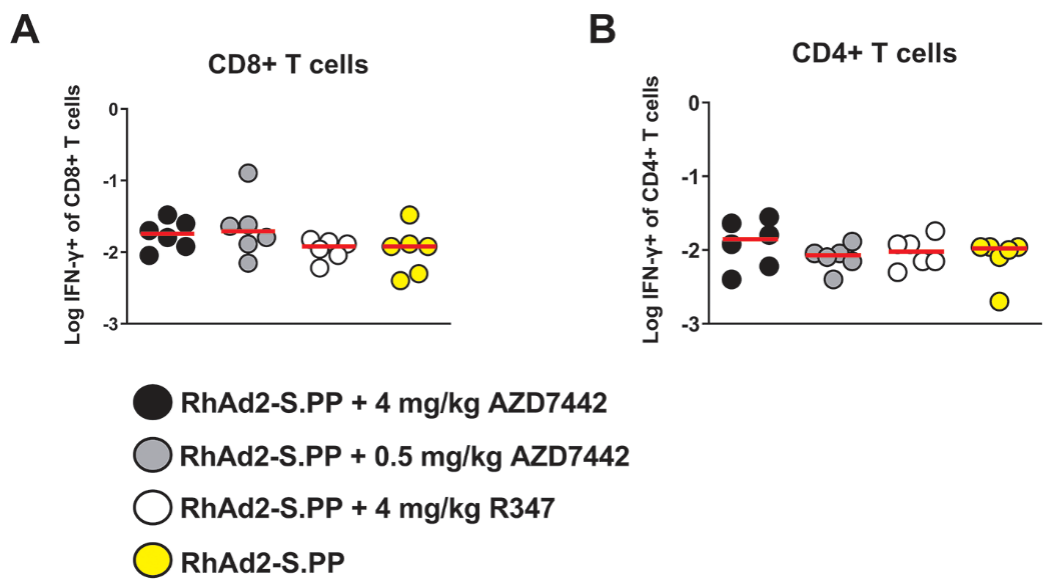
Vaccine-induced IFN-γ cellular immunity post SARS-CoV-2 viral-vector vaccine immunization is not impacted by AZD7442 mAb co-administration. S protein-specific (**A**) CD8+ and (**B**) CD4+ IFN-γ intracellular cytokine staining responses were quantified using peripheral blood mononuclear cells isolated at week 10 after vaccination (n=6 per group). Red horizontal bars indicate median values. A one-way ANOVA with Tukey correction was used for statistical analyses.

The potential impact of the AZD7442 mAbs on vaccine-elicited CD8+ and CD4+ cellular immune responses was assessed by interferon (IFN)-γ intracellular cytokine staining assays at week 10 using overlapping S peptides from the WA1/2020 strain. Comparable S protein-specific CD4+ and CD8+ T cell responses were observed in all groups receiving the vaccine, regardless of co-administration of the AZD7442 mAbs (P>0.05) (**Fig. 5** **; table S7**). These data show that vaccine-elicited IFN-γ mediated cellular immunity was not impacted by the co-administration of the AZD7442 mAb cocktail with the vaccine.

### There is no direct interaction between AZD7442 monoclonal antibody combination and RhAd52-S.PP vaccine

**Fig. 6.**
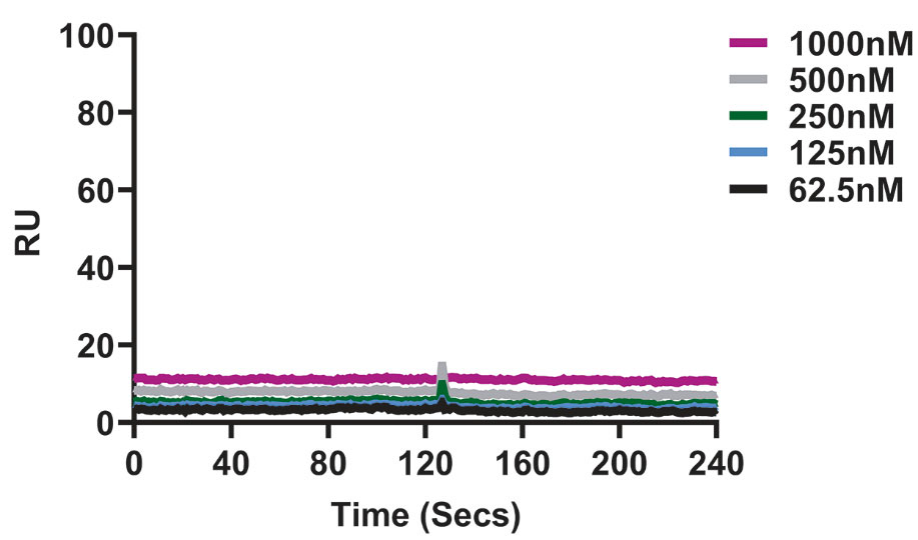
AZD7442 mAb does not bind to the RhAd52-S.PP vaccine. Binding of AZD7442 mAb cocktail to RhAd52-S.PP vaccine was determined by surface plasmon resonance. A titration series of AZD7442 was flowed over RhAd52-S.PP immobilized on a C1 sensor chip. RU indicates Response Units.

Finally, to assess if there was any direct binding between the AZD7442 mAbs and the RhAd52-S.PP vaccine, surface plasmon resonance assays were performed. A titration series of AZD7442 flowed over immobilized RhAd52-S.PP showed no observable binding **(** **Fig. 6** **; table S8)**. These data indicate that there was no notable interaction between AZD7442 mAb cocktail and the RhAd52-S.PP vaccine.

## DISCUSSION

Multiple vaccines and mAbs have been developed for the prevention of SARS-CoV-2 infection and development of COVID-19. It is likely that vaccines and mAbs may need to be used simultaneously in certain outbreak settings, and thus it is important to determine if one intervention may impact the other. In the current study, we demonstrate that administration of a bivalent combination of SARS-CoV-2 mAbs (*5-6, -8*) with a prototypic viral-vectored vaccine (*9*) did not lead to reduced potency of either intervention. Specifically, the SARS-CoV-2 mAbs did not diminish the kinetics, durability, or breadth of vaccine-induced humoral and cellular immunity.

The AZD7442 mAb cocktail retained some neutralization activity against the SARS-CoV-2 B.1.1.529 (Omicron) variant. This finding is in line with emerging data that shows that many SARS-CoV-2 mAbs lose substantial efficacy against B.1.1.529 (*11*). The AZD7442 mAb combination recently received emergency use authorization (EUA) by the US Food and Drug Administration (FDA) (*12*). The RhAd52.S-PP vaccine is currently not in clinical development but is analogous to the Ad26.COV2.S vaccine (*13*, *14*) in that both are recombinant adenovirus vector-based vaccines expressing the same stabilized SARS-CoV-2 S protein.

This study has several important limitations. First, the generalizability of our data to other vaccine platforms, including mRNA vaccines and adjuvanted protein vaccines, remains to be determined. It is possible that mAbs may impact protein-based vaccines more than gene-based vaccines. Second, although these current data show that SARS-CoV-2 mAbs can be co-administered with a vector-based vaccine without loss of potency of either product, further studies would need to assess any potential impact on protective efficacy. Third, important differences exist between macaques and humans, and thus clinical trials will be required to confirm these results. Nevertheless, the current data demonstrate that SARS-CoV-2 mAbs and an adenovirus vector-based vaccine can be co-administered in macaques.

## MATERIALS AND METHODS

### Study design

This study was designed to assess the impact of potent neutralizing SARS-CoV-2 mAbs on the immunogenicity of a genetic-based, prototypic viral vector-based SARS-CoV-2 vaccine when they are co-administered and was powered to show a 2-fold difference in immune responses. Cynomolgus macaques (*Macaca fascicularis*) (n=24; 12 female and 12 male) age range 2 to 7.5 years old were randomly allocated to 4 experimental groups (n=6 per group) and each animal immunized with 10^11^ viral particles of viral vector-based RhAd52-S.PP (*9*) by the intra-muscular route in the right quadriceps. On the same day and within 30 min of RhAd52-S.PP injections, animals received concurrent administration of 4, 0.5, or 0 mg/kg AZD7442 mAbs or 4 mg/kg isotype matched sham mAb R347 by the intra-muscular route in the left quadriceps. Blood samples were obtained in anesthetized macaques by the femoral vein. Peripheral blood mononuclear cells were isolated from anticoagulated blood and serum was processed from clotted blood. Assays were conducted blinded and no data were excluded.

All animals were subsequently assessed over a 36-week period for human IgG pharmacokinetics by ELISA, WA1/2020 neutralizing titers (with B.1.1.529 neutralizing titers additionally assessed at peak immunogenicity) by pseudovirus neutralization assays, and vaccine-induced antibody binding to parental (WA1/2020) and variant SARS-CoV-2 S protein and RBD antigens by multiplex electrochemiluminescence serology assays. Cellular immunity was assessed by intra-cellular cytokine staining at 10 weeks post-interventions. AZD7442 comprises a 1:1 equimolar concentrations of potent human SARS-CoV-2 neutralizing mAbs [tixagevimab (AZD8895) and cilgavimab (AZD1061)] (*6*, *8*). All mAbs were engineered for increased serum half-life and reduced Fc receptor and complement C1q binding. The facility where this non-human primate (NHP) study was conducted (Bioqual Inc.) is fully accredited by the Association for Assessment and Accreditation of Laboratory Animal Care International (AAALAC) and approved by the Office of Laboratory Animal Welfare (NIH/PHS Assurance ID: D16-00052). The study was conducted in compliance with all relevant local, state, and federal regulations and were approved by the Animal Care and Use Committee (IACUC) at Bioqual.

### Human IgG mAb ELISA

Animals were assessed over a 36-week period for human IgG mAb pharmacokinetics in serum using a previously described method (*5*)*.* In brief, ELISA plates were coated overnight at 4°C with 1 μg/mL of goat anti-human IgG (H+L) secondary antibody (monkey pre-adsorbed) (Novus Biologicals) and then blocked for 2 hours. The serum samples were assayed at 3-fold dilutions starting at a 1:3 dilution in Blocker Casein in PBS (Thermo Fisher Scientific) diluent. Samples were incubated for 1 hour at ambient temperature and then removed, and plates were washed. Wells then were incubated for 1 hour with horseradish peroxidase (HRP)-conjugated goat anti-Human IgG (monkey pre-adsorbed) (Southern Biotech) at a 1:4000 dilution. Wells were washed and then incubated with SureBlue Reserve TMB Microwell Peroxidase Substrate (Seracare) (100 μL/well) for 3 min followed by TMB Stop Solution (Seracare) to stop the reaction (100 μL/well). Microplates were read at 450 nm. The concentrations of the human mAbs were interpolated from the linear range of concurrently run purified human IgG (Sigma) standard curves using Prism software, version 8.0 (GraphPad).

### Pseudovirus neutralizing antibody assay

The SARS-CoV-2 pseudoviruses expressing a luciferase reporter gene were used to measure pseudovirus neutralizing antibodies (*10*). In brief, the packaging construct psPAX2 (AIDS Resource and Reagent Program), luciferase reporter plasmid pLenti-CMV Puro-Luc (Addgene) and S protein expressing pcDNA3.1-SARS-CoV-2 SΔCT were co-transfected into HEK293T cells (ATCC CRL_3216) with lipofectamine 2000 (Thermo Fisher Scientific). Pseudoviruses of SARS-CoV-2 variants were generated by using WA1/2020 strain (Wuhan/WIV04/2019, GISAID accession ID: EPI_ISL_402124) or B.1.1.529 (Omicron, GISAID ID: EPI_ISL_7358094.2). The supernatants containing the pseudotype viruses were collected 48 hours after transfection; pseudotype viruses were purified by filtration with 0.45-μm filters. To determine the neutralization activity of human serum, HEK293T cells expressing human angiotensin converting enzyme 2 (HEK293T-hACE2 cells) were seeded in 96-well tissue culture plates at a density of 2.0 × 10^4^ cells per well overnight. Three-fold serial dilutions of heat-inactivated serum samples were prepared and mixed with 50 μl of pseudovirus. The mixture was incubated at 37°C for 1 hour before adding to HEK293T-hACE2 cells. After 48 hours, cells were lysed in Steady-Glo Luciferase Assay (Promega) according to the manufacturer’s instructions. SARS-CoV-2 neutralization titers were defined as the sample dilution at which a 50% reduction (NT_50_) in relative light units (RLU) was observed relative to the average of the virus control wells.

### Enzyme-linked immunosorbent assay

SARS-CoV-2 RBD-specific binding antibodies in serum were assessed by ELISA. 96-well plates were coated with 1 μg/mL of SARS-CoV-2 WA1/2020 RBD protein in 1× Dulbecco phosphate-buffered saline (DPBS) and incubated at 4°C overnight. After incubation, plates were washed once with wash buffer (0.05% Tween 20 in 1× DPBS) and blocked with 350 μL of casein block solution (Thermo Fisher Scientific) per well for 2 to 3 hours at room temperature. Following incubation, block solution was discarded and plates were blotted dry. Serial dilutions of heat-inactivated serum diluted in casein block were added to wells, and plates were incubated for 1 hour at room temperature, prior to 3 more washes and a 1-hour incubation at room temperature in the dark with a 1μg/mL dilution of anti–macaque IgG HRP (Nonhuman Primate Reagent Resource AB_2819289) that does not cross-react with human IgG. Plates were washed 3 times, and 100 μL of SeraCare KPL TMB SureBlue Start solution was added to each well; plate development was halted by adding 100 μL of SeraCare KPL TMB Stop solution per well. The absorbance at 450 nm was recorded with a VersaMax microplate reader (Molecular Devices). For each sample, the ELISA end point titer was calculated using a 4-parameter logistic curve fit to calculate the reciprocal serum dilution that yields an absorbance value of 0.2. Interpolated end point titers were reported.

### Electrochemiluminescence assay (ECLA)

ECLA plates [Meso Scale Diagnostics (MSD) SARS-CoV-2] were designed and produced with up to 10 antigen spots in each well, including S proteins and RBDs from multiple SARS-CoV-2 variants. The plates were blocked with 50 μL of Blocker A (1% bovine serum albumin in distilled water) solution for at least 30 min at room temperature shaking at 700 rpm with a digital microplate shaker. During blocking, the serum was diluted to 1:5,000 or 1:50,000 in MSD Diluent 100. The calibrator curve was prepared by diluting the calibrator mixture from MSD 1:10 in Diluent 100 and then preparing a 7-step 4-fold dilution series plus a blank containing only MSD Diluent 100. The plates were then washed 3 times with 150 μL of Wash Buffer (0.5% Tween in 1x PBS), blotted dry, and 50 μL of the diluted samples and calibration curve were added in duplicate to the plates and set to shake at 700 rpm at room temperature for at least 2 hours. The plates were again washed 3 times and 50 μL of SULFO-Tagged anti-Human IgG detection antibody diluted to 1x in Diluent 100 was added to each well; samples were then incubated with shaking at 700 rpm at room temperature for at least 1 hour. Plates were then washed 3 times and 150 μL of MSD GOLD Read Buffer B was added to each well; the plates were read immediately after on a MESO QuickPlex SQ 120 machine. MSD titers for each sample was reported as Relative Light Units (RLU), which were calculated as Sample RLU minus Blank RLU and then fit using a logarithmic fit to the standard curve. The upper limit of detection was defined as 2x10^6^ RLU for each assay. If the signal exceeded this value at 1:5,000 serum dilution, the sample was run again at 1:50,000 and the fitted RLU was multiplied by 10 before reporting. The lower limit of detection was defined as 1 RLU and an RLU value of 100 was defined to be positive for each assay.

### Intra-cellular cytokine staining

CD4+ and CD8+ T cell responses were quantitated by pooled peptide-stimulated intracellular cytokine staining (ICS) assays. Peptide pools were 16 amino acid peptides overlapping by 11 amino acids spanning the SARS-CoV-2 WA1/2020 S protein (21st Century Biochemicals). 10^6^ peripheral blood mononuclear cells per well were re-suspended in 100 μL of R10 media(RPMI-1640 supplemented with 10% fetal bovine serum and penicillin-streptomycin) supplemented with CD49d monoclonal antibody (1 μg/mL) and CD28 monoclonal antibody (1 μg/mL). Each sample was stimulated with mock (100 μL of R10 plus 0.5% dimethyl sulfoxide; background control), peptides (2 μg/mL), or 10 pg/mL phorbol myristate acetate (PMA) and 1 μg/mL ionomycin (Sigma-Aldrich) (100μL; positive control) and incubated at 37°C for 1 hour. After incubation, 0.25 μL of GolgiStop (BD Biosciences) and 0.25 μL of GolgiPlug (BD Biosciences) in 50 μL of R10 was added to each well; samples were incubated at 37°C for 8 hours and then held at 4°C overnight. The next day, the cells were washed twice with DPBS, stained with aqua live/dead dye for 10 min and then stained with predetermined titers of monoclonal antibodies against CD279 (2 μL/test of clone EH12.1, brilliant blue 700, BD Pharmingen), CD38 [0.5 μL/test with clone OKT10, phycoerythrin (PE), NHP Reagent Resource], [ 2.5 μL/test with CD28 (clone 28.2, PE-Cy5), CD4 [ 0.625 μL/test with clone L200, brilliant violet (BV) 510, BD Pharmingen], CD95 [0.5 μL/test with clone DX2, brilliant ultraviolet (BUV) 737, BD Pharmingen], and CD8 (1 μL/test with clone SK1, BUV805, BD Pharmingen) for 30 min. Cells were then washed twice with 2% fetal bovine serum in DPBS buffer and incubated for 15 min with 200 μL of BD CytoFix/CytoPerm Fixation/Permeabilization solution (BD Biosciences). Cells were washed twice with 1X Perm Wash buffer (BD Perm/Wash Buffer 10X in the CytoFix/CytoPerm Fixation/Permeabilization kit diluted with MilliQ water and passed through 0.22μm filter) and stained intracellularly with monoclonal antibodies against Ki67 [1.25 μL/test with clone B56, fluorescein isothiocyanate (FITC), BD Pharmingen], CD69 (0.625 μL/test with clone TP1.55.3, ECD, Beckman Coulter), interleukin (IL)-10 (2 μL/test with clone JES3-9D7, PE-Cy7, BioLegend), IL-13 (2.5 μL/test with clone JES10-5A2, BV421, BD Pharmingen), tumor necrosis factor (TNF)-α (2.5 μL/test with clone Mab11, BV650, BD Pharmingen), IL-4 (2.5 μL/test with clone MP4-25D2, BV711, Pharmingen), IFN-γ (2.5 μL/test with clone B27; BUV395, BD Pharmingen), CD45 (0.05 μL/test with clone D058-1283, BUV615, BD Pharmingen), IL-2 [2.5 μL/test with clone MQ1-17H12, allophycocyanin (APC), BD Pharmingen], and CD3 (0.25 μL/test with clone SP34.2, Alexa 700, BD Pharmingen)for 30 min. Cells were washed twice with 1X Perm Wash buffer and fixed with 250μL of freshly prepared 1.5% formaldehyde. Fixed cells were transferred to 96-well round bottom plate and analyzed by BD FACSymphony system. Data were analyzed using FlowJo v9.9.

### Surface plasmon resonance binding assay

Assays were performed using a Biacore 3000 (Cytiva) and HBS-EP immobilization buffer (0.01 M HEPES pH 7.4, 0.15 M NaCl, 3 mM EDTA, 0.005% v/v Surfactant P20) (Cytiva). RhAd52-S.PP at 10^11^ viral particles was diluted 1:5 in acetate 4.5 buffer (Cytiva) and immobilized to approximately 500 response units (RU) on a C1 sensor chip using a standard amine coupling protocol. Multiple concentrations of AZD7442 (1000nM, 250nM, 500nM, 125nM and 62.5nM) were titrated over the sensor chip with an association time of 120 s and a dissociation time of 120 s at a flow rate of 50 μL/min. The surface was regenerated with a 30 s injection of 25mM NaOH at a flow rate of 50 μL/min. All sensogram plots were subtracted from the reference flow cell and buffer cycle to remove non-specific responses.

### Statistics

Verification of normal data distribution was conducted using the Shapiro-Wilk normality test. Determination of significance where required was conducted using a one-way analysis of variance (ANOVA) with Tukey’s correction for multiple comparisons. All statistics were performed using GraphPad prism software version 9.3.1

## Supplementary Materials

### Other Supplementary Material for this manuscript includes the following:

MDAR Reproducibility Checklist
