# Supplementary material for: A bivalent SARS-CoV-2 monoclonal antibody combination does not impact the immunogenicity of a vector-based COVID-19 vaccine in macaques
Source: Sci Transl Med. 2022 Jul 12:eabo6160. doi: 10.1126/scitranslmed.abo6160 (PMC9348747; doi:10.1126/scitranslmed.abo6160)
Supplement: Supplementary file 1 — Fig. S1 Tables S1 to S8 [file scitranslmed.abo6160_sm.pdf]

Science Translational Medicine
AAAS

Supplementary Materials for

A bivalent SARS-CoV-2 monoclonal antibody combination does not impact the immunogenicity of a vector-based COVID-19 vaccine in macaques

Joseph P. Nkolola *et al.*

Corresponding author: Dan H. Barouch, dbarouch@bidmc.harvard.edu

DOI: 10.1126/scitranslmed.abo6160

The PDF file includes:

Fig. S1
Tables S1 to S8

Other Supplementary Material for this manuscript includes the following:

MDAR Reproducibility Checklist

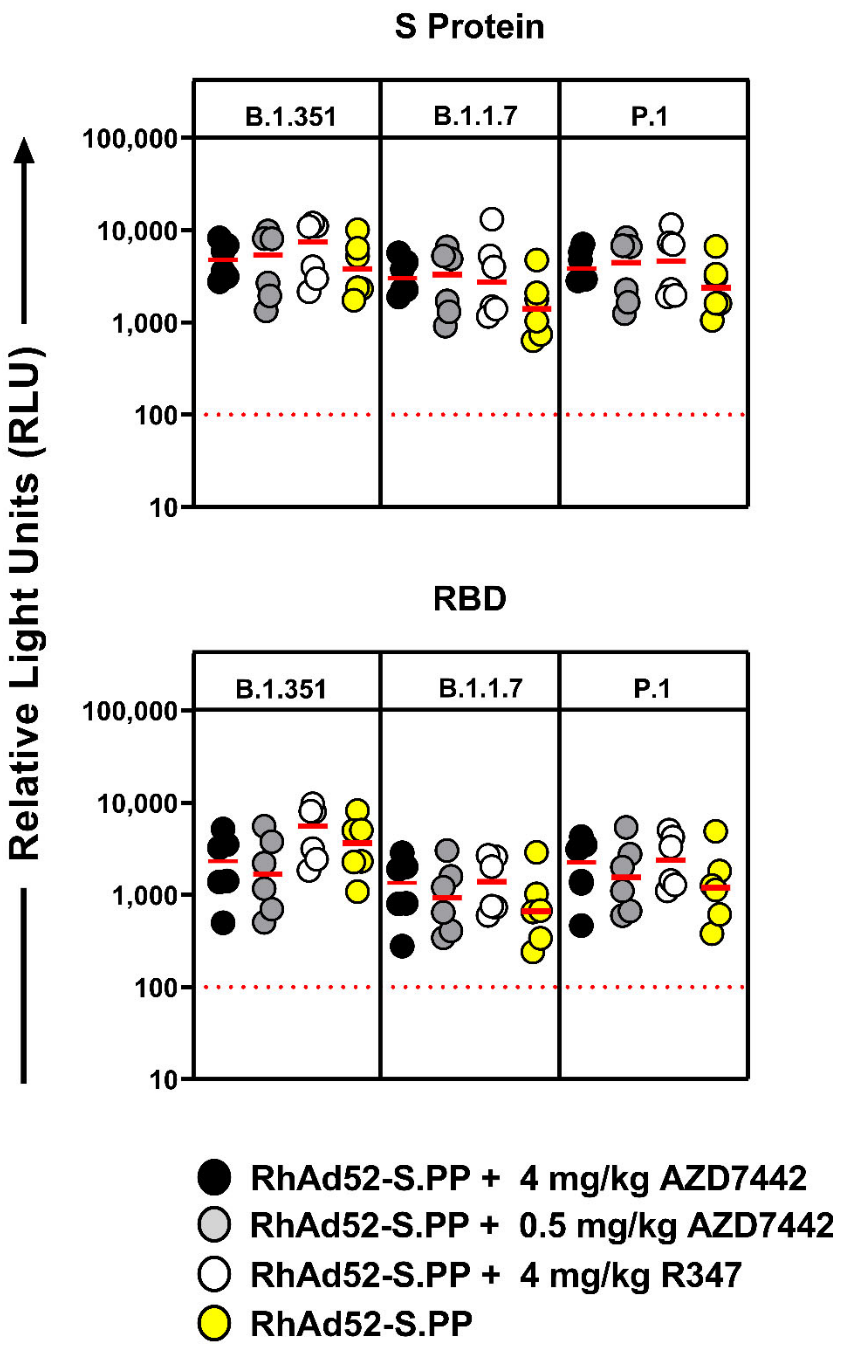

Figure S1. Extended Spike (S) and receptor binding domain (RBD) variant antigen multiplex IgG serology. Electrochemiluminesence multiplex IgG serology binding antibody responses against S protein and RBD B.1.351, B.1.1.7 and P.1 variant antigens at peak immunogenicity (Day 29) are shown. Solid red horizontal bars depict median quantities. Horizontal red dotted lines depict an arbitrarily defined assay positivity threshold of mean background + 2 standard deviations. Each data point is the mean of technical duplicates. Data were generated over several batch analyses of samples (N=1). Each data point is the mean

of technical duplicates. One-way analysis of variance (ANOVA) with Tukey's correction was used for statistical analyses.

Table S1. Raw AZD7442 pharmacokinetics; *indicates animal excluded due to high background at baseline. NHP, non-human primate; mAb, monoclonal antibody.

		mAb Concentration (µg/mL)														
**Experimental Group**	**NHP ID#**	**Baseline**	**Week 0 day 1**	**Week 0 day 3**	**Week 1 day 7**	**Week 2**	**Week 4 day 28**	**Week 6 day 42**	**Week8 day 56**	**Week 10 day 70**	**Week 12 day 84**	**Wk16 day 112**	**Week 20 day 140**	**Week 24 day 168**	**Week 28 day 196**	**Week 36 day 252**
**RhAd52-S.PP + 4 mg/kg AZD7442**	A18918	<0.01	44.0	59.4	38.9	23.9	17.6	10.9	8.5	2.7	3.8	0.7	0.3	0.1	0.06	<0.01
	18C5	<0.01	25.2	43.5	31.9	25.0	17.3	6.6	1.1	0.3	0.1	<0.01	<0.01	<0.01	<0.01	<0.01
	A20105	<0.01	28.8	61.2	41.0	23.1	12.3	10.2	9.4	5.5	3.9	0.7	0.6	0.2	0.10	0.04
	AA433IBB	<0.01	23.9	57.8	41.6	26.8	16.2	11.7	8.8	6.6	5.6	0.6	0.3	0.3	0.13	0.05
	AE575KC	<0.01	26.7	46.3	33.0	23.0	13.3	7.0	2.5	0.2	<0.01	<0.01	<0.01	<0.01	<0.01	<0.01
	AG195JB*	4.5	42.5	85.1	40.8	25.3	21.8	12.9	10.7	7.1	8.4	0.6	0.5	0.2	0.14	0.07
**RhAd52-S.PP + 0.5 mg/kg AZD7442**	AG208IC	<0.01	4.8	5.5	4.9	3.0	2.1	0.8	0.3	0.2	<0.01	<0.01	<0.01	<0.01	<0.01	<0.01
	AJ707HA	<0.01	4.9	6.5	3.6	2.9	1.4	0.9	0.3	0.4	0.1	<0.01	<0.01	<0.01	<0.01	<0.01
	AJ966DD	<0.01	2.5	5.7	2.8	1.8	0.04	<0.01	<0.01	<0.01	<0.01	<0.01	<0.01	<0.01	<0.01	<0.01
	AK506FC*	14.2	9.6	15.6	13.1	5.9	9.7	3.8	5.4	2.9	1.1	0.5	0.4	0.2	0.15	0.05
	AL176K	<0.01	1.9	4.3	3.3	3.2	2.8	0.8	<0.01	<0.01	<0.01	<0.01	<0.01	<0.01	<0.01	<0.01
	AM369AG	<0.01	3.1	5.9	3.3	1.1	2.0	0.9	0.7	0.7	0.4	0.2	0.1	0.05	0.04	<0.01
**RhAd52-S.PP + 4 mg/kg R347**	AM903BC	<0.01	42.6	58.1	54.7	44.5	32.1	18.5	15.6	9.1	10.6	4.4	1.5	0.7	0.24	0.16
	AN165M	<0.01	43.4	71.2	40.2	55.0	30.8	19.5	15.9	11.5	15.3	4.8	1.6	0.6	0.29	0.17
	AN29L	<0.01	46.0	68.5	45.7	37.5	28.4	15.9	12.3	7.1	8.2	3.5	1.1	0.4	0.15	0.06
	AN926L	<0.01	51.3	55.5	47.3	43.9	27.6	22.5	14.7	6.2	5.7	3.2	1.1	0.4	0.16	0.06
	AP162G	<0.01	69.5	83.9	46.5	40.1	30.2	23.4	17.1	14.3	15.5	4.8	2.1	0.7	0.35	0.12
	AP986D	<0.01	33.9	58.2	36.0	35.3	25.2	15.5	10.9	6.4	5.6	2.2	0.9	0.3	0.11	0.04
**RhAd52-S.PP**	AR446K	<0.01	<0.01	<0.01	<0.01	<0.01	<0.01	<0.01	<0.01	<0.01	<0.01	<0.01	<0.01	<0.01	<0.01	<0.01
	AR477M	<0.01	<0.01	<0.01	<0.01	<0.01	<0.01	<0.01	<0.01	<0.01	<0.01	<0.01	<0.01	<0.01	<0.01	<0.01
	AR947H	<0.01	<0.01	<0.01	<0.01	<0.01	<0.01	<0.01	<0.01	<0.01	<0.01	<0.01	<0.01	<0.01	<0.01	<0.01
	AT170J	<0.01	<0.01	<0.01	<0.01	<0.01	<0.01	<0.01	<0.01	<0.01	<0.01	<0.01	<0.01	<0.01	<0.01	<0.01
	AT291L	<0.01	<0.01	<0.01	<0.01	<0.01	<0.01	<0.01	<0.01	<0.01	<0.01	<0.01	<0.01	<0.01	<0.01	<0.01
	AT562C*	24.9	20.0	18.5	10.7	10.5	9.6	22.5	3.3	2.3	2.1	0.7	0.5	0.2	0.12	0.06

Table S2. Raw 50% neutralization titers (NT_{50}) against WA1/2020 pseudovirus; * indicates animal excluded due to high background at baseline.

	NT_{50} Titer																								
	RhAd52-S.PP + 4 mg/kg AZD7442						RhAd52-S.PP + 0.5 mg/kg AZD7442						RhAd52-S.PP + 4 mg/kg R347						RhAd52-S.PP					
NHP ID#	A18918	18C5	A20105	AA433IBB	AE575KC	AG195JB*	AG208IC	AJ707HA	AJ966DD	AK506FC*	AL176K	AM369AG	AM903BC	AN165M	AN29L	AN926L	AP162G	AP986D	AM903BC	AN165M	AN29L	AN926L	AP162G	AP986D*
Baseline	20	24	27	20	34	298	38	22	20	1198	28	20	20	20	20	20	23	20	20	20	20	20	25	1127
Week 1 day 7	13469	11073	14542	8784	9393	18389	925	470	1842	2270	815	1101	1101	58	115	185	148	53	32	159	31	69	56	1735
Week 2 day 14	15607	23447	11164	8805	7244	28972	1383	750	2171	8239	4341	1605	46	360	1146	454	106	113	104	3153	211	208	556	1391
Week 4 day 28	16185	3562	2470	4122	415	6956	269	129	20	2749	1104	48	111	164	654	178	21	96	97	637	140	138	213	1081
Week 6 day 42	3056	1265	1356	3630	826	10928	152	59	32	1100	227	55	155	119	239	201	92	40	74	345	165	47	152	604
Week 8 day 56	554	407	676	572	248	997	117	308	92	207	115	126	248	103	607	99	108	88	93	404	295	143	349	118
Week 10 day 70	263	91	170	987	82	689	112	116	115	222	117	54	165	47	54	97	75	30	43	235	74	67	119	128
Week 12 day 84	90	69	558	286	178	343	43	95	99	115	111	154	105	65	122	249	168	113	77	131	87	61	199	124
Week 16 day 112	84	73	143	279	161	889	65	146	284	144	56	317	142	149	151	115	103	37	49	196	113	124	249	132
Week 20 day 140	112	52	126	130	135	588	60	237	187	86	81	214	116	188	121	231	177	54	45	209	106	168	217	105
Week 24 day 168	77	75	123	94	140	698	88	139	175	91	74	228	107	101	226	108	72	42	44	105	102	105	198	161
Week 28 day 196	72	134	115	93	177	626	101	391	130	100	77	152	152	146	369	135	133	67	81	97	112	96	156	188
Week 36 day 252	124	82	102	71	105	570	60	159	189	79	54	98	124	123	259	88	139	76	51	186	94	99	172	251

Table S3. Raw NT_{50} B.1.1.529 (Omicron) pseudovirus neutralization titers at Day 7; * indicates animal excluded due to high background at baseline.

		NT_{50} Titer
**Experimental Group**	**NHP ID#**	**B.1.1.529**
**RhAd52-S.PP + 4 mg/kg AZD7442**	A18918	301.8
	18C5	226.9
	A20105	259.4
	AA433IBB	174.9
	AE575KC	142.0
	AG195JB*	204.5
**RhAd52-S.PP + 0.5 mg/kg AZD7442**	AG208IC	31.7
	AJ707HA	26.6
	AJ966DD	55.9
	AK506FC*	50.5
	AL176K	46.0
	AM369AG	32.3
**RhAd52-S.PP + 4 mg/kg R347**	AM903BC	20.0
	AN165M	20.0
	AN29L	20.0
	AN926L	20.0
	AP162G	20.0
	AP986D	20.0
**RhAd52-S.PP**	AR446K	20.0
	AR477M	20.0
	AR947H	20.0
	AT170J	20.0
	AT291L	20.0
	AT562C*	29.9

Table S4. Raw interpolated WA1-2020 RBD enzyme-linked immunosorbent assay (ELISA) binding antibody titers; * indicates animal excluded due to high background at baseline.

	Interpolated Binding Titer																							
	RhAd52-S.PP + 4 mg/kg AZD7442						RhAd52-S.PP + 0.5 mg/kg AZD7442						RhAd52-S.PP + 4 mg/kg R347						RhAd52-S.PP					
**NHP ID#**	**A18918***	**18C5**	**A20105**	**AA433IBB**	**AE575KC**	**AG195JB**	**AG208IC**	**AJ707HA**	**AJ966DD**	**AK506FC***	**AL176K**	**AM369AG**	**AM903BC**	**AN165M**	**AN29L**	**AN926L**	**AP162G**	**AP986D**	**AM903BC**	**AN165M**	**AN29L**	**AN926L**	**AP162G**	**AP986D***
**Baseline**	163.0	25.0	25.0	25.0	25.0	25.0	25.0	25.0	25.0	27.9	25.0	25.0	25.0	25.0	25.0	25.0	25.0	25.0	25.0	25.0	25.0	25.0	25.0	60.8
**Week 0 day 1**	162.2	36.9	25.0	25.0	45.8	51.3	25.0	25.0	25.2	38.8	31.7	25.0	25.0	25.0	25.0	25.0	25.0	25.0	25.0	25.0	25.0	25.0	25.0	65.2
**Week 0 day 3**	339.5	100.9	41.5	27.9	46.9	132.3	26.6	30.0	35.8	61.4	25.0	25.1	25.0	25.0	25.0	25.0	25.0	25.0	25.0	25.0	25.0	25.0	25.0	104.6
**Week 2 day 14**	569.5	4402.3	2866.1	142.2	2758.8	147.1	129.5	3884.0	298.4	912.3	284.0	1451.0	4515.2	7189.4	7623.9	7365.8	1012.0	2056.9	1136.1	3253.0	2701.1	2205.6	16870.1	883.5
**Week 4 day 28**	1553.8	6464.5	3769.9	363.4	4552.9	1806.0	521.4	4737.1	2806.2	1139.1	2096.9	2901.3	20677.9	10038.0	16237.0	16095.9	3424.3	10833.5	3626.3	34433.2	9649.7	7100.9	8625.4	3227.2
**Week 6 day 42**	1295.5	3712.6	2991.8	777.8	3951.5	2534.5	431.8	6186.7	6478.0	1071.3	1852.7	4148.0	6029.6	3960.6	9677.3	7965.4	3020.3	2258.0	1224.2	8643.6	8442.2	2049.8	6132.3	1805.3
**Week 8 day 56**	1991.5	3218.8	2896.7	1160.4	3534.6	4372.0	1465.1	6548.4	7622.0	1549.1	2322.2	5294.4	6062.6	3316.3	11007.8	5032.2	2787.8	2066.5	1609.4	9859.0	18879.4	1940.8	5519.8	1701.4
**Week 10 day 70**	1089.1	2194.6	2836.8	1629.5	2385.6	3534.4	1122.0	6004.1	4637.9	1316.0	2298.2	3265.0	3826.1	3368.8	6676.3	5006.2	1752.5	1548.6	1238.3	4850.0	4381.6	1936.9	3530.0	1660.8
**Week 12 day 84**	1335.0	2383.3	3396.4	1608.4	2174.3	3118.1	1430.3	4177.1	6922.7	1482.2	2458.4	4685.7	4695.8	4677.4	8912.2	5987.7	2403.2	1278.9	1946.6	4697.5	4987.5	2006.4	4654.5	1824.3
**Week 16 day 112**	805.4	1200.3	1735.7	1003.9	1511.8	2842.3	700.8	2127.5	3005.5	1297.6	1211.0	2234.1	2303.7	2680.2	3978.5	1837.0	1588.9	1137.2	1135.8	2869.9	2689.2	1650.9	3031.8	1518.3
**Week 20 day 140**	1285.4	1536.6	2022.2	1257.8	1763.8	3625.6	1078.2	2508.7	3857.0	1180.7	1246.7	3021.6	3178.1	2362.1	4187.5	3240.9	2281.4	1760.3	1356.1	3316.2	3784.3	2109.1	2972.2	2335.7
**Week 24 day 168**	1286.3	1533.1	1777.4	848.3	1309.7	4170.4	1266.4	2939.6	2698.1	1119.7	1089.2	1435.3	3296.2	2184.6	2857.5	2038.5	1343.0	1340.9	894.8	2894.3	3285.3	1023.4	1869.9	1653.8
**Week 28 day 196**	1089.9	1771.4	1583.6	566.9	907.4	4673.1	1010.0	2771.4	2460.2	809.2	1029.2	1395.2	2089.3	2187.8	4060.5	1758.2	1150.9	1237.2	648.5	3303.9	2575.6	1451.3	2367.9	2205.1
**Week 36 day 252**	846.3	759.4	1149.3	481.1	962.8	2470.1	728.1	1990.7	1869.4	727.3	1074.7	1794.2	1884.0	1881.0	2816.4	1519.2	1330.2	1010.6	607.6	2981.8	1974.4	1054.1	2447.5	2075.0

Table S5. Raw S protein electrochemiluminesence assay (ECLA) binding antibody response data.

			S protein					
		**Time point**	**Day 28**					
		**Virus strain**	WA1/2020	B.1.617.2	B.1.1.529	B.1.351	B.1.1.7	P.1
		**NHP ID#**						
**Experimental Group**	**RhAd52-S.PP + 4mg/kg AZD7442**	A18918	4541	2077	924	1884	2767	2938
		18C5	9077	4862	1820	4500	6866	5813
		A20105	10988	6068	2642	5674	8248	7059
		AA433IBB	4130	2480	1275	2256	3125	2917
		AE575KC	7298	4431	1915	3778	5948	4740
		AG195JB	4732	2825	1164	2252	3622	2829
	**RhAd52-S.PP + 0.5mg/kg AZD7442**	AG208IC	1729	1057	584	914	1332	1242
		AJ707HA	12448	7522	3274	6585	9834	8296
		AJ966DD	11129	5544	3100	4895	8136	6615
		AK506FC	3253	1939	1011	1711	2688	2270
		AL176K	2627	1508	734	1302	1941	1655
		AM369AG	10230	5678	3101	5257	8083	6798
	**RhAd52-S.PP + 4mg/kg R347**	AM903BC	24405	7908	2740	13156	11123	11478
		AN165M	6287	3208	742	1484	4010	2282
		AN29L	16537	9621	2733	5250	11972	7295
		AN926L	15968	8084	2160	3995	10966	6987
		AP162G	3064	1740	565	1162	2157	1893
		AP986D	4468	1936	504	1400	2996	1965
	**RhAd52-S.PP**	AR446K	3086	1941	289	633	2321	1052
		AR477M	12485	6078	1988	4751	10128	6666
		AR947H	8192	4086	928	1780	5227	3140
		AT170J	3411	1986	381	742	2382	1602
		AT291L	8875	4600	1269	2096	6383	3337
		AT562C	2175	1522	846	1035	1745	1616

Table S6 Raw RBD ECLA binding antibody response data.

			RBD					
		**Time point**	**Day 28**					
		**Virus strain**	WA1/2020	B.1.617.2	B.1.1.529	B.1.351	B.1.1.7	P.1
		**NHP ID#**						
**Experimental Group**	**RhAd52-S.PP + 4mg/kg AZD7442**	A18918	1634	1984	318	799	1379	1407
		18C5	6272	6397	926	2854	5180	4274
		A20105	4244	5424	818	1892	3234	3478
		AA433IBB	627	783	110	279	499	464
		AE575KC	4360	5424	1542	2010	3512	3113
		AG195JB	1778	2233	538	807	1410	1335
	**RhAd52-S.PP + 0.5mg/kg AZD7442**	AG208IC	644	802	182	342	503	594
		AJ707HA	7020	8310	1833	3025	5531	5384
		AJ966DD	4613	5278	817	1586	3777	2755
		AK506FC	945	1071	120	406	699	666
		AL176K	1324	1718	483	645	1167	1103
		AM369AG	2634	3363	729	1212	2200	2002
	**RhAd52-S.PP + 4mg/kg R347**	AM903BC	13181	9699	971	2596	7931	5017
		AN165M	5907	4857	171	599	3175	1105
		AN29L	13263	13812	1714	2680	9726	4236
		AN926L	12072	11926	694	2023	8076	3317
		AP162G	2471	2762	436	733	1845	1429
		AP986D	4489	2860	252	753	2424	1283
	**RhAd52-S.PP**	AR446K	3435	3103	92	240	2306	379
		AR477M	10036	8303	812	2895	8195	4896
		AR947H	7465	6923	678	1024	4993	1815
		AT170J	3248	3200	128	338	2263	611
		AT291L	8089	7217	297	654	5032	1261
		AT562C	1322	1600	601	676	1082	1131

Table S7. Raw CD4+ and CD8+ interferon (IFN)-γ intracellular cytokine staining cell populations (percentages).

		NHP ID#	Log IFN-γ of CD4+ T cells	Log IFN-γ of CD8+ T cells
Experimental Group	RhAd52-S.PP + 4mg/kg AZD7442	A18918	0.028	0.033
		18C5	0.023	0.02
		A20105	0.006	0.012
		AA433IBB	0.004	0.009
		AE575KC	0.012	0.016
		AG195JB	0.016	0.025
	RhAd52-S.PP + 0.5mg/kg AZD7442	AG208IC	0.009	0.023
		AJ707HA	0.004	0.024
		AJ966DD	0.009	0.127
		AK506FC	0.007	0.013
		AL176K	0.013	0.007
		AM369AG	0.008	0.016
	RhAd52-S.PP + 4mg/kg R347	AM903BC	0.012	0.014
		AN165M	0.018	0.013
		AN29L	0.007	0.015
		AN926L	0.007	0.011
		AP162G	0.012	0.006
		AP986D	0.005	0.009
	RhAd52-S.PP	AR446K	0.011	0.012
		AR477M	0.008	0.033
		AR947H	0.011	0.013
		AT170J	0.01	0.005
		AT291L	0.011	0.012
		AT562C	0.002	0.004

Table S8 Raw surface plasmon resonance binding reported in resonance units (RU).

AZD7442 Concentration (nM)	62.5	125	250	500	1000
Time (Secs)					
0	3.48	4.38	5.19	8.26	11.31
1	3.51	4.26	5.48	8.32	11.39
2	3.38	4.18	5.37	8.36	11.50
3	3.52	3.91	5.32	8.06	11.47
4	3.44	4.16	5.55	8.41	11.47
5	3.19	3.73	5.22	8.12	10.99
6	3.12	3.80	5.32	8.24	11.01
7	3.07	3.84	5.18	8.09	11.00
8	3.30	4.03	5.32	8.13	11.04
9	3.19	3.77	5.19	7.89	10.90
10	3.09	3.83	5.10	7.99	10.92
11	3.55	4.32	5.52	8.53	11.37
12	3.21	4.23	5.43	8.25	11.39
13	3.10	4.14	5.06	7.99	11.33
14	3.40	4.12	5.16	7.98	11.33
15	3.29	4.06	5.01	7.86	11.04
16	3.17	4.18	5.19	8.00	11.00
17	3.27	4.24	5.06	8.22	11.20
18	3.59	4.26	5.19	8.42	11.43
19	3.12	4.06	4.74	7.96	11.12
20	3.39	4.43	5.16	8.28	11.29
21	2.80	4.03	4.67	7.78	11.06
22	3.49	4.58	5.65	8.33	11.25
23	3.24	4.46	5.49	8.05	11.29
24	3.41	4.46	5.45	8.30	11.32
25	3.45	4.27	5.37	8.16	11.26
26	3.65	4.46	5.35	8.36	11.31
27	3.27	4.17	5.29	7.83	10.98
28	3.04	4.12	4.95	7.71	10.99
29	3.46	4.41	5.34	8.12	10.87
30	3.59	4.37	5.12	8.16	11.05
31	3.43	4.37	4.92	7.94	11.29
32	3.26	4.28	4.89	7.84	11.18
33	3.22	4.25	5.13	7.83	10.98
34	3.28	4.13	5.34	7.98	11.20
35	3.67	4.35	5.64	8.31	11.23
36	3.67	4.42	5.75	8.18	10.87
37	3.03	4.17	5.11	7.79	10.89
38	3.40	4.34	5.31	7.59	10.94
39	3.29	4.14	5.12	7.56	10.84
40	3.14	4.11	5.07	7.84	11.02
41	3.43	4.08	5.29	7.83	11.17
42	3.49	4.36	5.31	7.98	11.23
43	3.55	4.40	5.31	8.08	11.06
44	3.42	3.89	5.17	7.89	10.78
45	3.25	4.00	5.23	7.99	10.99
46	3.29	4.23	5.31	8.01	10.95
47	3.28	4.50	5.42	8.20	11.13
48	3.30	4.44	5.23	8.23	11.32
49	3.44	4.36	5.45	8.17	11.34
50	3.24	4.23	5.30	8.11	11.13
51	3.19	4.36	5.45	8.06	11.14
52	3.47	4.47	5.37	8.10	11.49
53	3.44	4.15	5.43	7.99	11.36
54	3.52	4.30	5.59	8.37	11.27
55	3.32	4.41	5.41	8.15	11.49
56	3.05	4.12	5.22	7.97	11.23
57	3.23	4.38	5.46	8.14	11.26
58	2.99	4.27	5.29	8.13	10.99
59	3.05	4.28	5.27	7.98	10.87
60	3.25	4.22	5.23	8.04	10.95
61	3.04	4.09	5.10	8.03	11.19
62	3.15	4.13	5.00	8.19	11.23
63	3.28	4.15	4.93	7.87	11.04
64	3.33	4.07	5.22	8.05	11.09
65	3.43	4.33	5.51	8.29	11.34
66	3.42	4.06	5.30	8.07	11.05
67	3.39	4.34	5.22	7.88	10.94
68	3.35	4.19	5.33	7.87	10.98
69	3.09	4.09	5.12	7.95	10.92
70	3.45	4.47	5.41	8.23	11.06
71	3.32	4.48	5.36	8.13	11.06
72	3.37	4.72	5.55	8.05	11.14
73	3.42	4.70	5.61	8.01	11.40
74	3.45	4.17	5.37	8.01	11.07
75	3.68	4.32	5.44	8.22	11.20
76	3.26	4.44	5.48	7.90	11.08
77	3.38	4.39	5.43	8.06	11.13
78	3.42	4.52	5.38	8.30	11.14
79	3.25	4.33	5.45	8.31	10.90
80	3.39	4.24	5.57	8.12	10.90

AZD7442 Concentration (nM)	62.5	125	250	500	1000
Time (Secs)					
81	3.51	4.51	5.58	8.14	10.84
82	3.56	4.32	5.39	7.92	10.66
83	3.60	4.36	5.44	7.83	10.79
84	3.87	4.71	5.66	8.22	11.25
85	3.90	4.63	5.37	8.02	11.05
86	3.90	4.62	5.67	8.10	11.16
87	3.63	4.50	5.49	7.90	11.00
88	3.92	4.54	5.65	8.11	11.35
89	3.52	4.75	5.88	8.15	11.39
90	3.58	4.24	5.77	8.08	11.07
91	4.01	4.37	5.55	8.24	11.45
92	3.74	4.32	5.58	7.93	11.26
93	3.70	4.19	5.56	8.03	11.21
94	3.70	4.03	5.72	7.97	11.18
95	3.83	4.59	5.63	8.34	11.21
96	3.76	4.37	5.60	8.17	11.01
97	3.46	4.50	5.64	8.14	11.38
98	3.38	4.58	5.69	8.18	11.27
99	3.81	4.81	5.97	8.43	11.42
100	4.09	4.80	6.10	8.57	11.57
101	3.94	4.65	5.92	8.36	11.39
102	3.74	4.62	5.94	8.40	11.25
103	4.02	4.75	5.83	8.52	11.42
104	3.99	4.74	5.77	8.46	11.70
105	3.34	4.58	5.45	7.99	11.23
106	3.43	4.54	5.47	8.13	11.44
107	3.39	4.55	5.69	8.25	11.49
108	3.13	4.46	5.57	8.26	11.43
109	3.11	4.36	5.53	8.18	11.41
110	3.21	4.15	5.39	7.91	11.30
111	3.14	4.19	5.41	7.73	10.97
112	3.27	4.49	5.58	7.84	11.18
113	3.10	4.26	5.23	7.67	10.97
114	3.37	4.49	5.53	8.09	11.32
115	3.31	4.25	5.47	7.90	11.03
116	3.04	4.30	5.54	7.84	11.12
117	3.36	4.46	5.62	8.16	11.30
118	3.15	4.32	5.42	8.16	11.04
119	3.29	4.52	5.52	8.18	11.38
120	3.76	4.81	5.73	8.45	11.54
121	3.43	4.49	5.52	8.16	11.27
122	3.36	4.44	5.40	8.18	11.09
123	3.48	4.51	5.69	8.24	11.01
124	3.71	4.71	5.72	8.27	11.21
125	3.75	4.58	5.66	8.23	11.04
126	3.81	4.43	5.68	8.24	11.28
127	4.96	5.64	9.60	14.47	11.80
128	3.35	4.29	6.52	9.14	11.37
129	3.50	4.38	5.74	7.97	11.53
130	3.37	4.16	5.17	7.65	11.55
131	3.81	4.18	5.28	7.70	11.67
132	3.79	4.21	5.29	7.76	11.66
133	3.47	4.11	5.05	7.70	11.54
134	3.13	3.74	4.78	7.58	11.27
135	3.01	3.91	4.84	7.68	11.26
136	3.02	3.94	4.97	7.41	11.38
137	3.11	4.01	5.03	7.39	11.24
138	3.17	3.92	5.00	7.32	11.31
139	3.14	3.86	5.27	7.35	11.42
140	3.13	3.67	5.04	7.45	11.30
141	3.28	3.82	5.06	7.34	11.48
142	2.93	3.58	4.79	7.05	11.22
143	2.94	3.56	4.73	7.18	11.12
144	2.89	3.67	4.53	7.03	11.09
145	2.72	3.66	4.51	7.27	11.08
146	2.73	3.76	4.69	7.10	11.09
147	2.78	3.76	4.60	7.33	11.16
148	3.00	3.72	4.82	7.51	11.05
149	2.84	3.44	4.71	7.26	10.82
150	2.61	3.29	4.55	7.08	10.87
151	2.96	3.59	4.57	7.40	11.24
152	2.88	3.82	4.56	7.36	10.92
153	2.78	3.53	4.58	7.17	10.98
154	2.89	3.67	4.68	7.18	11.21
155	2.67	3.61	4.71	7.28	11.09
156	2.51	3.26	4.38	7.01	10.89
157	2.61	3.59	4.49	7.02	10.91
158	2.77	3.78	4.63	6.89	11.13
159	2.78	3.48	4.51	6.85	11.22
160	2.71	3.77	4.75	7.11	11.44
161	3.25	4.02	4.94	7.26	11.45

AZD7442 Concentration (nM)	62.5	125	250	500	1000
Time (Secs)					
162	3.28	3.99	4.80	7.15	11.43
163	2.80	3.70	4.59	7.06	11.08
164	2.84	3.38	4.57	6.95	10.93
165	2.79	3.30	4.58	6.78	11.08
166	2.55	3.51	4.39	6.79	10.77
167	2.71	3.67	4.53	6.95	10.87
168	2.75	3.58	4.74	6.90	10.87
169	2.71	3.66	4.72	6.96	10.88
170	2.84	3.62	4.65	6.84	10.76
171	2.61	3.45	4.16	6.66	10.72
172	2.63	3.56	4.43	6.79	10.72
173	2.52	3.33	4.59	6.88	10.55
174	2.56	3.50	4.70	6.79	10.62
175	2.64	3.63	4.80	7.05	10.79
176	2.73	3.66	4.95	7.05	10.92
177	2.67	3.86	5.08	7.23	11.24
178	2.65	3.99	5.01	6.92	10.87
179	2.47	3.94	4.75	6.83	10.98
180	2.48	3.79	4.58	6.70	10.89
181	2.70	3.79	4.82	6.98	11.17
182	2.70	3.82	4.69	7.00	11.09
183	2.75	3.69	4.72	7.19	11.00
184	2.60	3.56	4.53	6.98	11.10
185	2.71	3.72	4.74	7.17	11.10
186	2.77	3.76	4.87	7.16	10.98
187	2.68	3.80	4.85	6.91	10.83
188	2.69	3.96	4.96	7.08	10.87
189	2.92	3.94	4.89	7.26	11.03
190	2.81	3.84	4.90	7.10	11.02
191	3.12	4.14	5.20	7.27	11.21
192	3.05	4.07	5.06	7.30	10.84
193	3.14	4.05	5.03	7.38	10.91
194	2.83	4.20	5.09	7.54	11.13
195	3.02	4.15	5.07	7.36	11.02
196	2.79	3.76	4.83	7.20	10.77
197	2.91	3.70	4.69	7.20	10.79
198	3.03	4.03	4.91	7.17	10.91
199	3.01	3.91	5.00	7.20	10.86
200	3.33	3.91	5.09	7.14	10.91
201	3.01	3.59	4.89	7.04	10.68
202	3.01	3.51	5.08	6.96	10.34
203	3.08	3.65	5.02	7.03	10.57
204	2.83	3.57	4.82	7.30	10.51
205	2.88	3.48	5.04	7.38	10.77
206	2.90	3.56	4.94	7.17	10.74
207	2.76	3.69	4.94	6.93	10.63
208	2.83	3.77	4.99	6.96	10.90
209	2.60	3.48	4.79	6.83	10.50
210	2.61	3.55	4.73	7.15	10.54
211	2.70	3.44	4.71	6.88	10.78
212	2.69	3.33	4.77	7.01	10.82
213	2.96	3.35	4.71	7.29	10.94
214	2.75	3.09	4.70	7.11	10.60
215	2.58	3.10	4.32	6.70	10.30
216	2.57	3.24	4.27	6.72	10.46
217	2.61	3.25	4.38	7.02	10.38
218	2.83	3.47	4.47	7.28	10.63
219	2.80	3.43	4.35	7.25	10.46
220	2.82	3.67	4.69	7.24	10.50
221	2.90	3.71	4.72	7.44	10.55
222	3.05	3.66	4.42	6.94	10.47
223	2.85	3.48	4.33	6.98	10.31
224	2.60	3.38	4.31	6.95	10.29
225	2.56	3.50	4.47	7.14	10.51
226	2.45	3.61	4.73	6.94	10.71
227	2.59	3.63	4.60	7.20	10.78
228	2.64	3.61	4.54	7.18	10.63
229	2.69	3.81	4.77	7.18	10.85
230	2.95	3.85	4.82	7.04	10.83
231	3.02	4.15	4.99	7.09	10.70
232	2.76	3.85	4.85	7.01	10.61
233	2.83	3.81	4.83	7.00	10.81
234	2.79	3.82	4.84	6.93	10.77
235	2.78	3.98	4.94	7.04	10.86
236	2.75	4.00	4.81	6.85	10.87
237	2.71	3.81	4.90	6.95	10.80
238	2.64	3.73	4.89	7.07	10.65
239	2.77	3.73	4.87	6.87	10.64
240	2.68	3.78	4.60	6.83	10.60
