# Supplementary material for: A bivalent SARS-CoV-2 monoclonal antibody combination does not impact the immunogenicity of a vector-based COVID-19 vaccine in macaques
Source: Sci Transl Med. 2022 Jul 12:eabo6160. doi: 10.1126/scitranslmed.abo6160 (PMC9348747; doi:10.1126/scitranslmed.abo6160)
Supplement: Supplementary file 2 — MDAR Reproducibility Checklist [file scitranslmed.abo6160_reproducibility_checklist.pdf]

Materials Design Analysis Reporting (MDAR) Checklist for Authors

The MDAR framework establishes a minimum set of requirements in transparent reporting applicable to studies in the life sciences (see Statement of Task: doi:10.31222/osf.io/9sm4x.). The MDAR checklist is a tool for authors, editors, and others seeking to adopt the MDAR framework for transparent reporting in manuscripts and other outputs. Please refer to the MDAR Elaboration Document for additional context for the MDAR framework.

For all that apply, please note where in the manuscript the required information is provided.

Materials:

Newly created materials	indicate where provided: page no/section/legend)	n/a
The manuscript includes a dedicated "materials availability statement" providing transparent disclosure about availability of newly created materials including details on how materials can be accessed and describing any restrictions on access.	Page 21/Data materials and availability	

Antibodies	indicate where provided: page no/section/legend)	n/a
For commercial reagents, provide supplier name, catalogue number and RRID, if available.	Anti-rhesus IgG1/3 [1B3]-HRP antibody, NIH Nonhuman Primate Reagent Resource Cat# PR-1234, RRID:AB_2819289 (Page 11/Materials & Methods)<br><br>Goat anti-Human IgG (H+L) Secondary Antibody (Monkey Pre-adsorbed)(Novus Biological, Cat No. NB7487) (Page 11/Materials & Methods)<br><br>Goat Anti-Human IgG HRP (Monkey ads) (Southern Biotech, Cat no. 2049-05) (Page 11/Materials & Methods)<br><br>BD Horizon BB700 Mouse Anti-Human CD279 (PD-1) (BD Pharmingen, Cat No: 566460) (Page 14/Materials & Methods)<br>38PE (NHP Reagent Resource, RRID: AB_2819278) (Page 14/Materials & Methods)<br>BD Pharmingen PE-Cy™5 Mouse Anti-Human CD28 (BD Pharmingen, Cat No: 555730) (Page 14/Materials & Methods)<br>BD Horizon BUV737 Mouse Anti-Human CD95 (BD Pharmingen, Cat No: 612790) (Page 14/Materials & Methods)<br>BD Horizon BV510 Mouse Anti-Human CD4 (BD Pharmingen, Cat No: 563094) (Page 14/Materials & Methods)<br>BD Horizon BUV805 Mouse Anti-Human CD8 (BD Pharmingen, Cat No: 612889) (Page 14/Materials & Methods)<br>BD Pharmingen Alexa Fluor 488 Mouse anti-Ki-67 (BD Pharmingen, Cat No:558616) (Page 15/Materials & Methods)<br>69 ECD (Beckman Coulter, Cat No: 6607110) (Page 15/Materials & Methods)<br>IL10 PE CY7 (Biolegend, Cat No: 501419) (Page 15/Materials & Methods)<br>BD Horizon BV421 Rat Anti-Human IL-13 (BD Pharmingen, Cat No:563580) (Page 15/Materials & Methods)<br>BD Horizon BV650 Mouse Anti-Human TNF (BD Pharmingen, Cat No: 563418) (Page 15/Materials & Methods)<br>BD Horizon BV711 Rat Anti-Human IL-4 (BD Pharmingen, Cat No: 564112) (Page 15/Materials & Methods)<br>BD Horizon BUV395 Mouse Anti-Human IFN-γ (BD Pharmingen, Cat No: 565971) (Page 15/Materials & Methods)<br>BD Pharmingen PE Mouse Anti-NHP CD45 (BD Pharmingen, Cat No: 552833) (Page 15/Materials &	

	Methods)<br>BD Pharmingen APC Rat Anti-Human IL-2 (BD Pharmingen, Cat No: 554567) (Page 15/Materials & Methods)<br>BD Pharmingen Alexa Fluor 700 Mouse Anti-Human CD3 (BD Pharmingen, Cat No: 557917) (Page 15/Materials & Methods)	

DNA and RNA sequences	**indicate where provided: page no/section/legend)**	**n/a**
**Short novel DNA or RNA including primers, probes:** Sequences should be included or deposited in a public repository.		N/A

Cell materials	**indicate where provided: page no/section/legend**	**n/a**
**Cell lines:** Provide species information, strain. Provide accession number in repository **OR** supplier name, catalog number, clone number, **OR** RRID.	HEK293T cells (ATCC, CRL-3216)	N/A
**Primary cultures:** Provide species, strain, sex of origin, genetic modification status.		N/A

Experimental animals	**indicate where provided: page no/section/legend)**	**n/a**
**Laboratory animals or Model organisms:** Provide species, strain, sex, age, genetic modification status. Provide accession number in repository **OR** supplier name, catalog number, clone number, **OR** RRID.		N/A
**Animal observed in or captured from the field:** Provide species, sex, and age where possible.	Macaca fascicularis, 12 female & 12 Male,<br>Age range 2-7.5 years	

Plants and microbes	**indicate where provided: page no/section/legend)**	**n/a**
**Plants:** provide species and strain, ecotype and cultivar where relevant, unique accession number if available, and source (including location for collected wild specimens).		N/A
**Microbes:** provide species and strain, unique accession number if available, and source.		N/A

Human research participants	**indicate where provided: page no/section/legend) or state if these demographics were not collected**	**n/a**
If collected and within the bounds of privacy constraints report on age, sex and gender or ethnicity for all study participants.		N/A

Design:

**Study protocol**	**indicate where provided: page no/section/legend)**	**n/a**
If study protocol has been pre-registered, provide DOI. For clinical trials, provide the trial registration number **OR** cite DOI.		N/A

**Laboratory protocol**	**indicate where provided: page no/section/legend)**	**n/a**
Provide DOI **OR** other citation details if detailed step-by-step protocols are available.		N/A

**Experimental study design (statistics details)**		
**For in vivo studies:** State whether and how the following have been done	**indicate where provided: page no/section/legend. If it could have been done, but was not, write not done**	**n/a**
Sample size determination	The study was powered to show a 2-fold difference in immune responses (Page 9/Study Design)	
Randomisation	Page 9/ Study design	
Blinding		N/A
Inclusion/exclusion criteria		N/A

**Sample definition and in-laboratory replication**	**indicate where provided: page no/section/legend**	**n/a**
State number of times the experiment was replicated in laboratory.	Single experiments were performed for all assays.	
Define whether data describe technical or biological replicates.	Technical replicates	

**Ethics**	**indicate where provided: page no/section/legend**	**n/a**
**Studies involving human participants:** State details of authority granting ethics approval (IRB or equivalent committee(s), provide reference number for approval.		N/A
**Studies involving experimental animals:** State details of authority granting ethics approval (IRB or equivalent committee(s), provide reference number for approval.	Page 10/Study design	
**Studies involving specimen and field samples:** State if relevant permits obtained, provide details of authority approving study; if none were required, explain why.		N/A

**Dual Use Research of Concern (DURC)**	**indicate where provided: page no/section/legend**	**n/a**
If study is subject to dual use research of concern regulations, state the authority granting approval and reference number for the regulatory approval.		N/A

Analysis:

Attrition	indicate where provided: page no/section/legend	n/a
Describe whether exclusion criteria were preestablished. Report if sample or data points were omitted from analysis. If yes report if this was due to attrition or intentional exclusion and provide justification.		N/A

Statistics	indicate where provided: page no/section/legend	n/a
Describe statistical tests used and justify choice of tests.	Page 16/Statistics. Data were verified to have normal distribution via Shapiro-Wilkes normaility test. Comparison of >2 groups groups was performed using one-way ANOVA with Tukey's correction for multiple comparisons.	

Data availability	indicate where provided: page no/section/legend	n/a
For newly created and reused datasets, the manuscript includes a data availability statement that provides details for access or notes restrictions on access.		N/A
If newly created datasets are publicly available, provide accession number in repository **OR** DOI **OR** URL and licensing details where available.		N/A
If reused data is publicly available provide accession number in repository **OR** DOI **OR** URL, **OR** citation.		N/A

Code availability	indicate where provided: page no/section/legend	n/a
For all newly generated custom computer code/software/mathematical algorithm or re-used code essential for replicating the main findings of the study, the manuscript includes a data availability statement that provides details for access or notes restrictions.		N/A
If newly generated code is publicly available, provide accession number in repository, **OR** DOI **OR** URL and licensing details where available. State any restrictions on code availability or accessibility.		N/A
If reused code is publicly available provide accession number in repository **OR** DOI **OR** URL, **OR** citation.		N/A

Reporting

MDAR framework recommends adoption of discipline-specific guidelines, established and endorsed through community initiatives. Journals have their own policy about requiring specific guidelines and recommendations to complement MDAR.

**Adherence to community standards**	**indicate where provided: page no/section/legend**	**n/a**
State if relevant guidelines (e.g., ICMJE, MIBBI, ARRIVE) have been followed, and whether a checklist (e.g., CONSORT, PRISMA, ARRIVE) is provided with the manuscript.		N/A
